# Use of computed tomography-derived body composition to determine the prognosis of patients with primary liver cancer treated with immune checkpoint inhibitors: a retrospective cohort study

**DOI:** 10.1186/s12885-022-09823-7

**Published:** 2022-07-06

**Authors:** Lu-shan Xiao, Rui-ning Li, Hao Cui, Chang Hong, Chao-yi Huang, Qi-mei Li, Cheng-yi Hu, Zhong-yi Dong, Hong-bo Zhu, Li Liu

**Affiliations:** 1 Big Data Center, Nanfang Hospital, Southern Medical University, Guangzhou, 510515 China; 2 Department of Infectious Diseases, Nanfang Hospital, Southern Medical University, Guangzhou, 510515 China; 3 Department of Infectious Diseases, Guangzhou First People’s Hospital, Guangzhou, 510515 China; 4 Department of Radiation Oncology, Nanfang Hospital, Southern Medical University, Guangzhou, 510515 China; 5 Department of Oncology, the First Affiliated Hospital, Hengyang Medical School, University of South China, Hengyang, 421001 China

**Keywords:** Primary liver cancer, Immune checkpoint inhibitors, Sarcopenia, Fat tissue, Prognosis

## Abstract

**Background:**

Immune checkpoint inhibitors (ICIs) have been used to successfully treat primary liver cancer (PLC); however, identifying modifiable patient factors associated with therapeutic benefits is challenging. Obesity is known to be associated with increased survival after ICI treatment; however, the relationship between body composition (muscle, fat) and outcomes is unclear. This study aimed to evaluate the association between sarcopenia and CT-derived fat content and the prognosis of ICIs for the treatment of PLC.

**Methods:**

In this retrospective cohort study of 172 patients with PLC, we measured the skeletal muscle index (SMI), skeletal muscle density, visceral adipose tissue index, subcutaneous adipose tissue index, total adipose tissue index (TATI), and visceral-to-subcutaneous adipose tissue area ratio using CT. In addition, we analyzed the impact of body composition on the prognosis of the patients. Multivariate Cox regression analysis was used to screen for influencing factors.

**Results:**

Among the seven body composition components, low SMI (sarcopenia) and low TATI were significantly associated with poor clinical outcomes. Multivariate analysis revealed that sarcopenia (hazard ratio [HR], 5.39; 95% confidence interval [CI], 1.74–16.74; *p* = 0.004) was a significant predictor of overall survival (OS). Kaplan–Meier curves showed that sarcopenia and TATI were significant predictors of OS. Body mass index was not associated with survival outcomes.

**Conclusions:**

Sarcopenia and fat tissue content appear to be independently associated with reduced survival rates in patients with PLC treated with ICIs.

**Supplementary Information:**

The online version contains supplementary material available at 10.1186/s12885-022-09823-7.

## Background

Primary liver cancer (PLC) is a common malignant tumor worldwide [1]. The number of global new cases of primary liver cancer increased from 471,000 in 1990 to 905,677 in 2020, and the incidence rate continues to increase [2]. Recently, immune checkpoint inhibitors (ICIs) have been used as a new treatment method for PLC, resulting in good anti-tumor activity and preliminary survival benefits [3–5]. Therefore, the focus of the current work is to determine which patients are most likely to respond to ICIs and identify modifiable patient factors associated with treatment benefits. Recent epidemiological studies have widely recognized that obesity is associated with improved response to ICIs in multiple cancer types and has an impact on the ICI dosing strategy [6].

Body mass index (BMI; calculated as weight in kilograms divided by height in meters squared) is a simple anthropometric index and is widely used. The relationship between obesity and primary liver cancer has been reported [7]. However, BMI has limitations. BMI does not assess body composition such as muscle volume or regional fat distribution, which may have a differing relationship with survival. Several recent studies have reported that sarcopenia and fat tissue, rather than BMI, are independent risk factors for the efficacy of ICIs in a variety of tumors. One study of patients with melanoma treated with ICIs reported that obesity and lower muscle quantity and quality were associated with poor prognosis [8]. Shiroyama et al. have found that baseline sarcopenia was significantly associated with poor survival outcomes in patients with advanced non-small cell lung cancer treated with ICIs [9]. However, the associations of muscle quantity, muscle quality, and fat distribution with ICI efficacy have not been confirmed. Therefore, we examined the relationship between body composition (including sarcopenia and fat distribution) and the clinical outcomes of a group of patients with PLC treated with ICIs.

## Methods

### Patient population

**Fig. 1:**
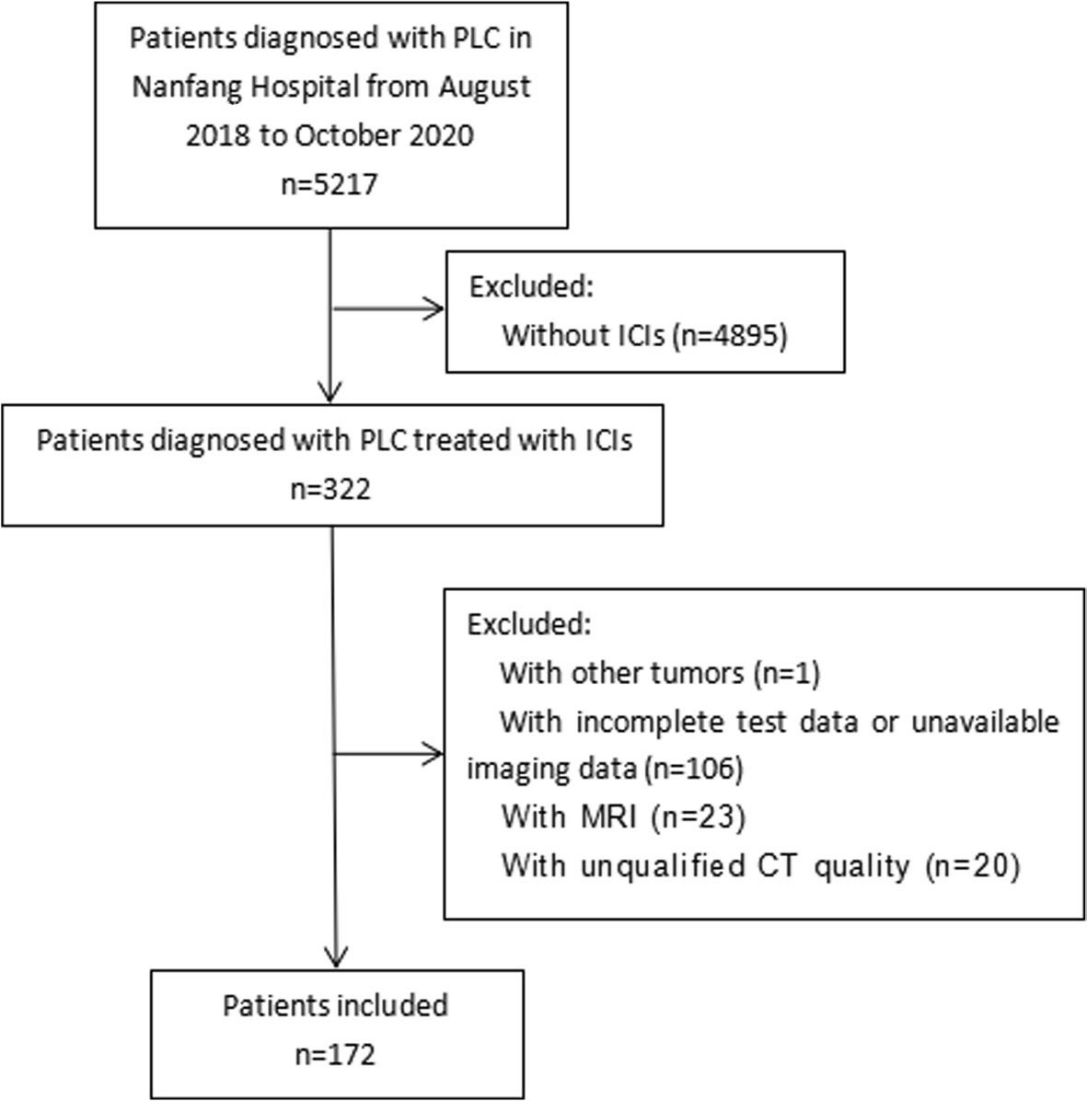
Flowchart of the study patient selection process. PLC primary liver cancer, ICI immune checkpoint inhibitor, CT computed tomography, MRI Magnetic Resonance Imaging

This retrospective cohort study included the data of patients with PLC treated with ICIs at Nanfang Hospital between August 2018 and October 2020. Treatment with ICI includes anti-programmed cell death 1 (anti-PD-1) antibodies (nivolumab, pembrolizumab, sintilimab, and tislelizumab) and anti-programmed cell death ligand-1 (anti-PD-L1) antibodies (atezolizumab, durvalumab, and avelumab). The patients underwent abdominal CT within 1 month before immunotherapy. We excluded patients with incomplete test data or unavailable imaging data, patients with magnetic resonance imaging, patients with CT images of insufficient quality, and patients with other types of tumors (Fig. 1). Progression-free survival (PFS) and overall survival (OS) were determined based on a review of the electronic medical records. For the patients who were seen last but had missing dates of death in the clinical records, we conducted a telephone follow-up, and those who were not contacted were recorded as lost. PFS was defined as the time from treatment initiation to progression, according to the Response Evaluation Criteria in Solid Tumors version 1.1. OS was defined as the time from treatment initiation to death or the last follow-up.

### Automatic segmentation

We quantified the data from a cross-sectional unenhanced phase CT image taken solely for the purpose of diagnosing and staging PLC (Additional file 1). The muscle area, muscle radiodensity, and adiposity were measured using CT scans obtained within 1 month before immunotherapy. Two senior radiologists quantified the cross-sectional area (in cm^2^) of muscle and adipose tissue at the third lumbar vertebra (L3); furthermore, they used SliceOmatic software (version 5.0; Tomovision, Montreal, Canada) to differentiate components based on tissue-specific ranges measured in Hounsfield units (HU) (Additional file 2). The single-slice abdominal cross-sectional area of the L3 vertebrae is closely related to the volume of whole-body muscle, whereas adipose tissue has been used in several previous studies [10–12]. Muscle tissue was identified as tissue with a radiodensity between − 29 and + 150 HU. The radiodensity of organs is within this range; hence, we combined the L3 muscles to divide the area to avoid mistakenly marking the organs as muscle tissue. Subcutaneous adipose tissue was defined as tissue located outside the boundary of the muscle area with radiation density between − 190 and − 30 HU. Visceral adipose tissue is the tissue located within the boundary of the muscle area with radiation density between − 150 and − 50 HU [13]. The subcutaneous adipose tissue and visceral adipose tissue were identified by automatic recognition of threshold by SliceOmatic combined with manual recognition of boundary.

### Body composition measurements

According to the European Consensus on the definition and diagnosis of sarcopenia, sarcopenia is defined by low measure levels for three parameters: (1) muscle strength, (2) muscle quantity/quality, and (3) physical performance as an indicator of severity [14]. However, this definition lacks clinical practicability. For the convenience of clinical use, we used SMI to evaluate sarcopenia, which has been widely used in previous studies [8, 10, 11]. The SMI was calculated as follows: skeletal muscle area (cm^2^)/height (m^2^) [15]. For patients with BMI < 25, male sarcopenia was defined as SMI < 43 cm/m^2^ and female sarcopenia as < 41 cm/m^2^. For patients with BMI ≥26, male sarcopenia was defined as < 53 cm/m^2^ and female sarcopenia as < 41 cm/m^2^ [15]. Skeletal muscle density (SMD) was measured by obtaining the average HU of the muscles at the L3 level. Several studies have shown that SMD is negatively correlated with myosteatosis, which is further associated with survival among patients with cancer [16, 17]. For patients with BMI < 24.9, low SMD was defined as < 41 HU. For patients with BMI > 25, low SMD was defined as < 33 HU [18, 19]. The visceral adipose tissue index (VATI) was calculated as the visceral adipose tissue area (cm^2^)/height (m^2^). The subcutaneous adipose tissue index (SATI) was calculated as the subcutaneous adipose tissue area (cm^2^)/height (m^2^). The total adipose tissue index (TATI) was the sum of SATI and TATI [8]. Because there is no clinically established threshold for the visceral adipose tissue index, SATI, VATI, and TATI, we used the receiver operating characteristic (ROC) curve to calculate the cut-off values for them. The visceral to subcutaneous adipose tissue area ratio (VSR) was calculated as the visceral adipose tissue area (cm^2^)/subcutaneous adipose tissue area (cm^2^). Males were divided into high and low VSR according to a cut-off value of 1.33, and the female cut-off value was 0.93 [11].

### Body mass index

BMI was calculated as weight (kg)/height (m^2^). According to the World Health Organization definition, BMI < 18.5 is considered underweight, BMI of 18.5–24.9 is considered normal weight, and BMI ≥25 is considered overweight [20].

### Statistical analysis

The t-test or non-parametric Mann–Whitney U test was used to compare continuous variables between groups. Pearson’s chi-squared test or Fisher’s exact test was used to compare categorical variables between groups. ROC curve analysis was performed to analyze the area under the ROC curve, and the Youden Index was used to identify the optimal cut-off values for VATI, SATI, and TATI. A multivariate Cox regression analysis was used to further screen for influencing factors. Kaplan–Meier curves were used to evaluate OS and PFS, which were compared between groups using the log-rank test. All statistical tests were two-tailed, and *p* < 0.05 was considered statistically significant. Multivariate Cox regression models were developed to estimate the survival of patients based on sarcopenia and fat tissue, and adjusted for covariates, including age, sex, Eastern Cooperative Oncology Group performance status (ECOG PS), Barcelona Clinic Liver Cancer (BCLC) staging system, Child–Pugh class, baseline metastasis, and previous treatment. All statistical analyses were performed using IBM SPSS software (version 26.0; IBM Corporation, Armonk, NY, USA).

## Results

### Patient characteristics

Of the 322 patients with PLC treated with ICIs admitted to our department between June 2018 and October 2020, 106 (32.9%) were excluded because of insufficient CT imaging and/or test data. We excluded 1 patient who had other tumors (0.3%), 23 patients who underwent magnetic resonance imaging (7.1%), and 20 patients with unqualified CT (6.2%). Therefore, we retrospectively assessed 172 patients (53.4%).

The mean patient age was 51.4 ± 11.7 years, and most (86.6%) patients were males. BCLC stage distribution was as follows: stage B, 36 (20.9%) patients, and stage C, 136 (79.1%) patients. Most patients had cirrhosis (59.3%), Child–Pugh class A (80.2%), ECOG-PS score of 0 (53.5%), and hepatitis B virus (89.0%). Furthermore, 78.5% of the patients had metastasis at baseline. The median follow-up duration was 9 months. Other baseline characteristics are shown in Table 1.

**Table 1:** Baseline characteristics of patients with PLC treated with ICIs

Characteristic	All patients (*N* = 172)
Age, mean ± SD	51.4 ± 11.7
Gender male, N (%)	149(86.6)
Body mass index (kg/m2), median (IQR)	22.2(20.0–24.2)
< 18.5 (underweight), N (%)	22(12.8)
18.5 ~ 24.9 (normal weight), N (%)	113(65.7)
≥ 25 (overweight), N (%)	37(21.5)
Viral status, N (%)	
HCV/HBV/HCV + HBV/none	3/153/2/14(1.7/89.0/1.2/8.1)
Alcohol consumption, N (%)	
> 80 g per day	19(11.0)
Smoking status, N (%)	
Never/former/current	101/19/52(58.7/11.0/30.2)
BCLC stage, N (%)	
B/C	36/136(20.9/79.1)
ECOG-PS, N (%)	
0/1/2/3	92/68/9/3(53.5/39.5/5.2/1.8)
Child-Pugh Class, N (%)	
A/B/C	138/32/2(80.2/18.6/1.2)
Comorbidity, N (%)	
Hypertension (yes/no)	35/137(20.3/79.7)
Diabetes (yes/no)	20/152(12.2/87.8)
Liver Cirrhosis (yes/no)	102/70(59.3/40.7)
Previous treatment (yes/no), N (%)	154/18(89.5/10.5)
Baseline metastasis (yes/no), N (%)	135/37(78.5/21.5)
Body composition variable	
SMI (cm^2^/m^2^), median (IQR)	46.0(41.1–52.5)
SMD (HU), median (IQR)	39.9(36.4–44.0)
VATI (cm2/m2), median (IQR)	29.0(15.1–46.6)
SATI (cm2/m2), median (IQR)	33.7(19.0–51.4)
TATI (cm2/m2), median (IQR)	66.9(35.2–99.6)
VSR, median (IQR)	0.84(0.59–1.19)

*PLC* Primary liver cancer, *ICI* Immune checkpoint inhibitor, *SD* Standard deviation, *IQR* Interquartile range, *HCV* Hepatitis s C virus, *HBV* Hepatitis B virus, *BCLC* Barcelona Clinic Liver Cancer, *ECOG* Eastern Cooperative Oncology Group, *PS* Performance status, *SMI* Skeletal muscle index, *SMD* Skeletal muscle density, *SMG* Skeletal muscle gauge, *VATI* Visceral adipose tissue index, *SATI* Subcutaneous adipose tissue index, *TATI* Total adipose tissue index, *VSR* Visceral to subcutaneous fat area ratio

### Prevalence of sarcopenia and factors associated with it

The overall prevalence of sarcopenia was 39.5%. The clinicopathological characteristics of patients with or without sarcopenia are summarized in Table 2. Significant differences were found between patients with or without sarcopenia regarding gender (*p* = 0.002), BMI (*p* = 0.002), viral status (*p* = 0.036), liver cirrhosis (*p* = 0.020), and baseline metastasis (*p* = 0.033). No significant difference was found between patients with or without sarcopenia as regards age, alcohol consumption, smoking status, BCLC stage, ECOG-PS, Child–Pugh Class, hypertension, diabetes, or previous treatment.

**Table 2:** Clinicopathological characteristics in the patients with or without sarcopenia

	Sarcopenia (*N* = 68)	Non sarcopenia (*N* = 104)	*p* value
Age, mean ± SD	51.8 ± 12.2	51.2 ± 11.3	0.729
Gender, N (%)			0.002
Male	52(76.5)	97(93.3)	
Female	16(23.5)	7(6.7)	
Body mass index (kg/m2), median (IQR)	20.5(18.3–24.2)	22.7(21.0–24.2)	0.002
Viral status, N (%)			0.036
HCV	1(1.5)	2(1.9)	
HBV	56(82.3)	95(91.4)	
HCV + HBV	0(0.0)	2(1.9)	
None	11(16.2)	5(4.8)	
Alcohol consumption, N (%)			0.452
> 80 g per day	6(8.8)	13(12.5)	
Smoking status, N (%)			0.261
Never	45(66.2)	56(53.9)	
former	7(10.3)	12(11.5)	
current	16(23.5)	36(34.6)	
BCLC stage, N (%)			0.392
B	12(17.6)	24(23.1)	
C	56(82.4)	80(76.9)	
ECOG-PS, N (%)			0.594
0	33(48.5)	59(56.7)	
1	29(42.7)	39(37.5)	
2	4(5.9)	5(4.8)	
3	2(2.9)	1(1.0)	
Child-Pugh Class, N (%)			0.266
A	54(79.4)	84(80.8)	
B	12(17.7)	20(19.2)	
C	2(2.9)	0(0.0)	
Comorbidity, N (%)			
Hypertension (yes/no)	17/51(25.0/75.0)	18/86(17.3/82.7)	0.221
Diabetes (yes/no)	10/58(14.7/85.3)	10/94(9.6/90.4)	0.309
Liver Cirrhosis (yes/no)	33/35(48.5/51.5)	69/35(66.3/33.7)	0.020
Previous treatment (yes/no), N (%)	60/8(88.2/11.8)	94/10(90.4/9.6)	0.653
Baseline metastasis (yes/no), N (%)	59/9(86.8/13.2)	76/28(73.1/26.9)	0.033

*SD* Standard deviation, *IQR* Interquartile range, *HCV* Hepatitis s C virus, *HBV* Hepatitis B virus, *BCLC* Barcelona Clinic Liver Cancer, *ECOG* Eastern Cooperative Oncology Group, *PS* Performance status

### Associations with body mass index

The univariate and multivariate analyses did not indicate any significant differences between BMI and OS or PFS (Additional file 3). Kaplan–Meier curves showed that the prognosis for underweight patients was worse than that for normal weight and overweight patients; however, the difference was not statistically significant (Additional file 4).

### Association with body composition measures

We used univariate and multivariate analyses to assess the associations among sarcopenia, fat content, and prognosis (Table 3). According to the univariate analysis, sarcopenia was a prognostic factor for OS (hazard ratio [HR], 4.90; 95% confidence [CI], 2.52–9.51; *p* < 0.001) and PFS (HR, 1.62; 95% CI, 1.18–2.28; *p* = 0.005). The OS of patients with a high VATI was better (HR, 0.30; 95% CI, 0.15–0.59; *p* = 0.001) than that of patients with a low VATI; however, no difference was noted in PFS. Similarly, the OS of patients with a high SATI and of those with a high TATI were better than those of patients with a low SATI (HR, 0.31; 95% CI, 0.17–0.58; *p* < 0.001) and those with a low TATI (HR, 0.31; 95% CI, 0.17–0.57; *p* < 0.001); however, no difference was observed in PFS. We did not find any statistically significant associations between VSR and OS or PFS.

**Table 3:** Univariable and multivariable analyses examining OS and PFS in association with body composition measures (*n* = 172)

Variable	Univariate analysis			Multivariate analysis*		
	HR	95% CI	P value	HR	95% CI	*P* value
**OS**						
Age (≥60 years)	1.23	0.63–2.41	0.550			
Gender male	0.46	0.22–0.97	0.042	2.39	0.59–9.63	0.222
BMI						
Underweight	2.13	0.96–4.75	0.063	1.00	0.21–4.84	0.998
Obesity	0.92	0.40–2.12	0.839	0.79	0.18–3.48	0.750
HBV	0.81	0.34–1.92	0.631			
Alcohol consumption (yes vs no)	0.19	0.03–1.40	0.103			
Smoking status (yes vs no)	1.06	0.54–2.07	0.872			
BCLC stage (C)	0.94	0.45–1.98	0.878			
ECOG-PS						
1	1.41	0.74–2.69	0.294	0.59	0.20–1.76	0.344
2	1.17	0.27–5.03	0.834	0.00	0.00	0.987
3	2.40	0.56–10.34	0.241	0.62	0.08–4.88	0.647
Child-Pugh Class						
B	2.19	1.09–4.39	0.028	1.68	0.55–5.15	0.367
C	7.69	1.02–57.9	0.048	12.43	0.91–170.39	0.059
Comorbidity						
Hypertension (yes vs no)	1.10	0.52–2.31	0.801			
Diabetes (yes vs no)	0.97	0.40–2.32	0.941			
Liver Cirrhosis (yes vs no)	0.78	0.42–1.44	0.421			
Previous treatment (yes vs no)	0.79	0.33–1.89	0.598			
Baseline metastasis (yes vs no)	1.23	0.57–2.65	0.609	0.59	0.21–1.67	0.323
CRP (≥10 mg/L)	2.80	1.12–7.02	0.028	0.53	0.16–1.72	0.289
PCT (≥1 ng/mL)	0.04	0.00–142.19	0.448			
NLR (≥2.57) 3	2.01	1.08–3.56	0.028	4.63	1.41–15.19	0.012
Body composition variable						
Sarcopenia	4.90	2.52–9.51	< 0.001	5.39	1.74–16.74	0.004
SMD (low vs high)	1.64	0.89–3.03	0.116	1.06	0.33–3.39	0.920
VATI (high vs low)	0.30	0.15–0.59	0.001	0.04	0.00–0.46	0.009
SATI (high vs low)	0.31	0.17–0.58	< 0.001	3.44	0.32–37.31	0.310
TATI (high vs low)	0.31	0.17–0.57	< 0.001	1.32	0.14–12.60	0.808
VSR (high vs low)	0.73	0.29–1.86	0.509			
**PFS**						
Age (≥60 years)	0.94	0.64–1.38	0.748			
Gender male	0.79	0.49–1.25	0.307	0.70	0.33–1.47	0.341
BMI						
Underweight	1.37	0.83–2.28	0.220	0.93	0.37–2.38	0.882
Obesity	1.06	0.71–1.59	0.766	0.59	0.28–1.24	0.162
HBV	1.33	0.80–2.22	0.267			
Alcohol consumption (yes vs no)	0.72	0.41–1.25	0.238			
Smoking status (yes vs no)	1.14	0.81–1.62	0.456			
BCLC stage (C)	1.26	0.84–1.88	0.265			
ECOG-PS						
1	1.24	0.89–1.75	0.205	1.25	0.76–2.05	0.387
2	1.93	0.96–3.88	0.064	1.36	0.34–5.38	0.665
3	0.75	0.18–3.06	0.690	1.70	0.34–8.58	0.519
Child-Pugh Class						
B	1.12	0.73–1.71	0.612	1.55	0.86–2.76	0.143
C	4.44	1.08–18.30	0.039	2.91	0.59–14.32	0.190
Comorbidity						
Hypertension (yes vs no)	0.99	0.66–1.49	0.953			
Diabetes (yes vs no)	0.98	0.61–1.59	0.941			
Liver Cirrhosis (yes vs no)	1.08	0.78–1.50	0.652			
Previous treatment (yes vs no)	1.44	0.83–2.49	0.195			
Baseline metastasis (yes vs no)	1.56	1.05–2.32	0.027	1.41	0.81–2.43	0.221
CRP (≥10 mg/L)	0.94	0.61–1.43	0.768	1.03	0.61–1.75	0.991
PCT (≥1 ng/mL)	0.80	0.36–1.77	0.588			
NLR (≥2.57)	1.07	0.77–1.49	0.676	1.21	0.73–1.99	0.460
Body composition variable						
Sarcopenia	1.62	1.18–2.28	0.005	1.48	0.82–2.67	0.195
SMD (low vs high)	0.83	0.60–1.16	0.269	0.56	0.32–0.99	0.044
VATI (high vs low)	0.87	0.53–1.43	0.590	1.07	0.37–3.11	0.895
SATI (high vs low)	0.80	0.54–1.18	0.259	1.28	0.37–4.47	0.695
TATI (high vs low)	0.90	0.61–1.33	0.590	0.67	0.21–2.12	0.491
VSR (high vs low)	0.98	0.63–1.52	0.937			

*OS* Overall survival, *PFS* Progression- free survival, *HR* Hazard ratio, *CI* Confidence interval, *BMI* Body mass index, *HBV* Hepatitis B virus, *BCLC* Barcelona Clinic Liver Cancer, *ECOG* Eastern Cooperative Oncology Group, *PS* Performance status, *CRP* c-reactive protein, *PCT* Procalcitonin, *NLR* Neutrophil-to-lymphocyte ratio, *SMD* Skeletal muscle density, *SMG* Skeletal muscle gauge, *VATI* Visceral adipose tissue index, *SATI* Subcutaneous adipose tissue index, *TATI* Total adipose tissue index, *VSR* Visceral to subcutaneous fat area ratio

*Adjusted for sex, BMI, ECOG PS score, Child–Pugh score, baseline metastasis, CRP, and NLR

Various studies have reported a negative relationship between ICI treatment and inflammation. Some inflammatory markers, including neutrophil-to-lymphocyte ratio (NLR), c-reactive protein (CRP), and procalcitonin (PCT), can predict the prognosis of patients treated with ICI. Therefore, we added CRP (≥10 mg/L), PCT (≥1 ng/mL), and NLR (≥2.57, according to median NLR) to our multivariable analyses [21–23]. According to the multivariate analysis (Table 3), after adjusting for sex, BMI, ECOG PS score, Child–Pugh score, baseline metastasis, CRP, and NLR, the OS of patients with sarcopenia was worse than that of patients with no sarcopenia (HR, 5.39; 95% CI, 1.74–16.74; *p* = 0.004); however, no significant difference was found in PFS between patients with sarcopenia and no sarcopenia (HR, 1.48; 95% CI, 0.82–2.67; *p* = 0.195). Additionally, the patients with low VATI had a better OS than those who with high VATI (HR, 0.04; 95% CI, 0.00–0.46; *p* = 0.009), and the patients with low SMD had a better PFS than those with high SMD (HR, 0.56; 95% CI, 0.32–0.99; *p* = 0.044).

**Fig. 2:**
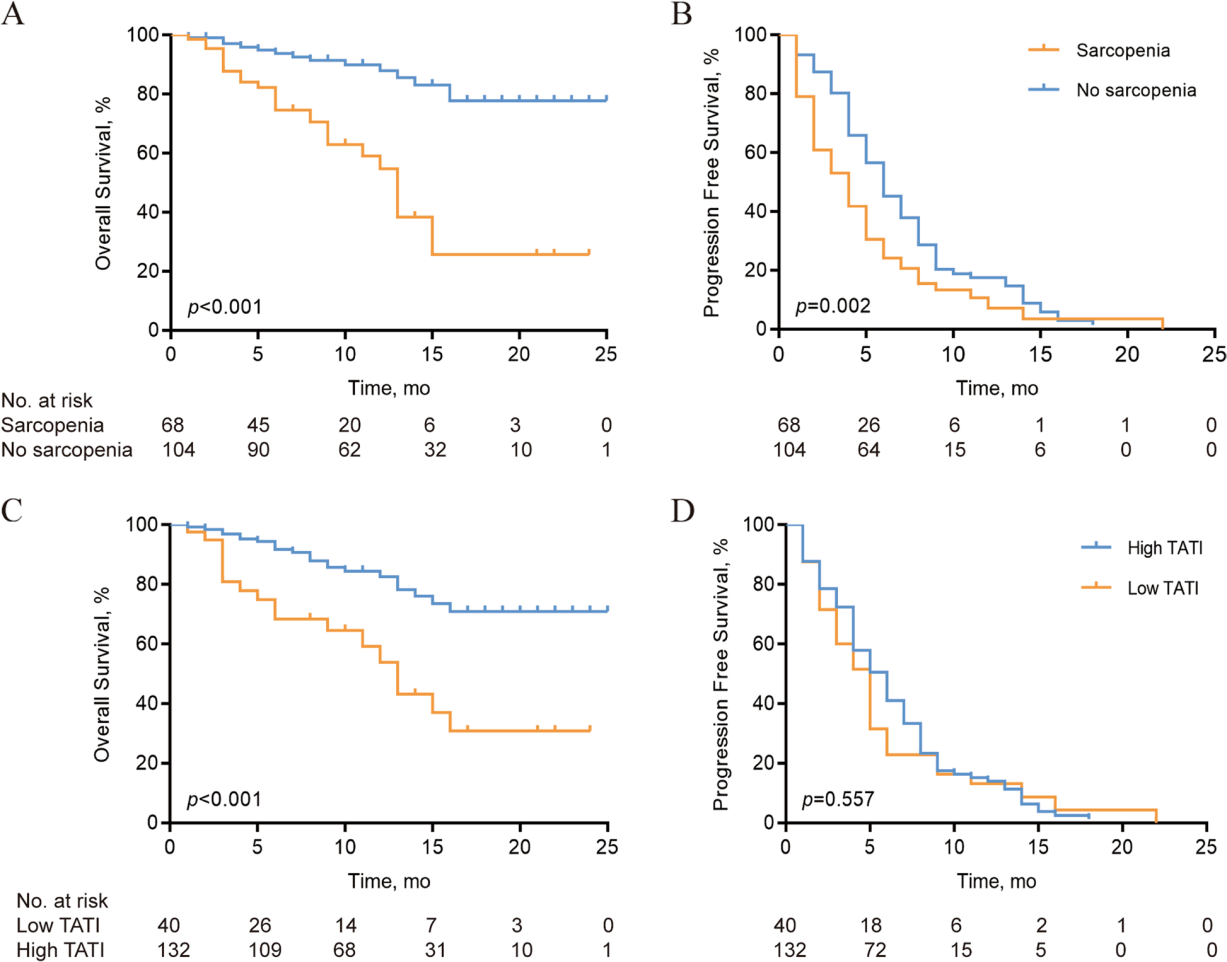
Survival outcomes for body composition. **a** Kaplan- Meier curves for OS for SMI. **b** Kaplan- Meier curves for PFS for SMI. **c** Kaplan- Meier curves for OS for TATI. **d** Kaplan- Meier curves for PFS for TATI. OS, overall survival; PFS, progression- free survival; SMI, skeletal muscle index; TATI, total adipose tissue index

Kaplan–Meier curves demonstrated that patients with sarcopenia and a low TATI had worse OS than those without sarcopenia (log-rank *p* < 0.001) and a high TATI (log-rank *p* < 0.001) (Fig. 2).

To further investigate the relationship between sarcopenia and fat content, we assessed different combinations of SMI and TATI (Additional file 5). When comparing cohorts with the poorest outcomes (low SMI:low TATI) to those with the best outcomes (high SMI:high TATI), there was a significant difference in both PFS and OS, with patients in the low SMI:low TATI group having significantly worse outcomes (*p* < 0.001 and *p* = 0.020 respectively, Additional file 5).

## Discussion

In this study, we verified that sarcopenia is associated with poor OS in patients with PLC treated with ICIs. Akce et al. have studied 57 patients with advanced PLC who received anti-PD1 antibody treatment and found that sarcopenia did not significantly predict OS [24]. On the contrary, we suggest that sarcopenia is an independent prognostic factor for patients with PLC treated with ICIs and that this could be proven by enrolling more patients in a similar study. However, we do not think that this hypothesis is specific to ICIs. Sarcopenia is a powerful prognostic factor for patients with liver cancer undergoing arterial embolization, chemotherapy, and hepatectomy [25–27]. Some studies have reported that sarcopenia is a prognostic factor of TKI, including sorafenib, in the treatment of HCC, which may be related to the effect of sarcopenia on inflammatory states and the immune microenvironment [28–30]. In PLC, significant differences were found between patients with or without sarcopenia regarding gender, BMI, viral status, liver cirrhosis, and baseline metastasis; however, they were not independent prognostic factors, which suggests that sarcopenia surpasses these factors as a prognostic effector. In addition, we found that sarcopenia, rather than low SMD, was associated with a poor prognosis, suggesting that the quantity rather than quality of muscle played a major role in the prognosis.

Moreover, we found that a low VATI was associated with poor OS for patients with PLC treated with ICIs, indicating that lower fat content is not conducive to survival in these patients. To our knowledge, our study is the first to reveal that higher fat content indicated on CT is independently associated with better survival in patients treated with ICIs. Previous studies have shown that high BMI is conducive to the survival of patients; however, BMI cannot distinguish between fat and muscle, and it cannot accurately reflect the body fat distribution [31–33]. During this study, we used CT to distinguish fat and muscle tissue and accurately measure the fat area, which is a strong proof that having fat tissue is an independent prognostic factor for patients with PLC treated with immunotherapy.

In this study, no statistically significant association was found between baseline BMI and the prognosis for ICI treatment. This is inconsistent with previous studies [19, 31] and may be due to a few reasons. First, previous studies have shown that the impact of BMI may be sex-specific; however, we did not conduct a sex-stratified analysis because of the small number of female patients (only 23). Second, patients with PLC always have ascites. Their non-muscle and non-fat tissues have an impact on BMI; therefore, BMI cannot accurately reflect the degree of obesity of these patients. However, by studying different BMI groups, we found that underweight patients tended to have worse OS than normal weight and overweight patients. This was consistent with our findings that a low TATI and sarcopenia were associated with poor OS and that insufficient muscle quantity and insufficient adipose tissue may lead to decreased BMI.

This study had several limitations. First, this was a retrospective study; therefore, subsequent clinical trials must be conducted to verify these results. Second, we could not explore the impact of sex on the results because few women were included in the study population. Third, our sample size was small; thus, it is necessary to conduct statistically powered large sample research in the future. Finally, patients who did not undergo CT or whose CT scans were not analyzable were not included in the study, which may have introduced bias.

## Conclusions

We did not find a significant association between BMI and the prognosis of patients with PLC treated with ICIs. However, we did find that patients with lower fat content and lower muscle quantity had a worse prognosis. This suggests that body composition is crucial for patients with PLC treated with ICIs. In clinical practice, the prognosis of patients with less fat and muscle may be poor. However, nutritional support, proper exercise, and the use of drugs to prevent muscle consumption may improve the survival of patients with PLC.

## Supplementary Information

**Additional file 1: Table S1.** The scan characteristics and image acquisition process.

**Additional file 2: Fig. S1.** Representative segmentation results. We used three colors to label adipose tissue and muscle tissue. Yellow = visceral adipose tissue, Blue = subcutaneous adipose tissue, Red = muscle.

**Additional file 3: Table S2.** Univariable and multivariable analysis examining OS, and PFS in association with BMI. The univariate and multivariate analyses did not indicate any significant differences between BMI and OS, or PFS.

**Additional file 4: Fig. S2.** Survival outcomes for body mass index. (a) Kaplan- Meier curves for OS. (b) Kaplan- Meier curves for PFS. OS, overall survival; PFS, progression- free survival. The figure showed that the prognosis for underweight patients was worse than that for normal weight and overweight patients; however, the difference was not statistically significant.

**Additional file 5: Fig. S3.** Kaplan- Meier curves for PFS and OS for various combinations of SMI:TATI. (a) Kaplan- Meier curves for OS. (b) Kaplan- Meier curves for PFS. OS, overall survival; PFS, progression- free survival SMI, skeletal muscle index; TATI, total adipose tissue index. We compared cohorts with the poorest outcomes (low SMI:low TATI) to those with the best outcomes (high SMI:high TATI) and found that there was a significant difference in both PFS and OS with patients in the low SMI:low TATI group having significantly worse outcomes (*p* < 0.001 and *p* = 0.020 respectively).

**Additional file 6.** STROBE Statement—checklist of items that should be included in reports of observational studies.

## Data Availability

The datasets generated and/or analyzed during the current study are not publicly available due the hospital policy and data privacy protection, but are available from the corresponding author on reasonable request.

## Abbreviations

BCLC: Barcelona Clinic Liver Cancer
BMI: Body mass index
CRP: C-reactive protein
ECOG-PS: Eastern Cooperative Oncology Group performance status
ICIs: Immune checkpoint inhibitors
NLR: Neutrophil-to-lymphocyte ratio
OS: Overall survival
PCT: Procalcitonin
PFS: Progression-free survival
PLC: Primary liver cancer
SATI: Subcutaneous adipose tissue index
ROC: Receiver operating characteristic
SMD: Skeletal muscle density
SMI: Skeletal muscle index
TATI: Total adipose tissue index
VATI: Visceral adipose tissue index
VSR: Visceral-to-subcutaneous adipose tissue area ratio
