# Supplementary material for: Use of computed tomography-derived body composition to determine the prognosis of patients with primary liver cancer treated with immune checkpoint inhibitors: a retrospective cohort study
Source: BMC Cancer. 2022 Jul 6;22:737. doi: 10.1186/s12885-022-09823-7 (PMC9258103; doi:10.1186/s12885-022-09823-7)
Supplement: Supplementary file 1 — Additional file 1: Table S1. The scan characteristics and image acquisition process. [file 12885_2022_9823_MOESM1_ESM.docx]

Additional file 1.

Table S1. **The scan characteristics and image acquisition process.**

All patients from the Nanfang Hospital, Southern Medical University underwent contrast-enhanced CT (CECT) using either of two multi-detector row CT (MDCT) systems: the SOMATOM (Siemens Medical Systems) or the Brilliance iCT 256 (Philips Healthcare). The scan characteristics are listed in the below Table. Additionally, we injected contrast material (1.5 mL/Kg, Ultravist 370, Bayer Schering Pharma) intravenously at a flow rate of 2.0–3.0 mL/s using a pump injector (Ulrich CT Plus 150, Ulrich Medical) to obtain CECT images. Four-phase (unenhanced, hepatic arterial, portal venous, and delayed phases) CT images were obtained at 0 s, 30 s, 60 s, and 120 s after injection, respectively.

| Parameter | SOMATOM | Brilliance iCT256 |
| --- | --- | --- |
| Tube voltage (kVp) | 120 | 120 |
| Tube current (mA) | Auto | Auto |
| Detector collimation (mm) | 64×0.6 | 128×0.625 |
| Field of view (mm) | 250–500 | 300-400 |
| Matrix size | 512×512 | 512×512 |
| Rotation times (s) | 0.5 | 0.5 |
| Slice interval (mm) | 0 | 0 |
| Slice thickness (mm) | 1-5 | 1-5 |
