# Supplementary figures and images for: Use of computed tomography-derived body composition to determine the prognosis of patients with primary liver cancer treated with immune checkpoint inhibitors: a retrospective cohort study
Source: BMC Cancer. 2022 Jul 6;22:737. doi: 10.1186/s12885-022-09823-7 (PMC9258103; doi:10.1186/s12885-022-09823-7)

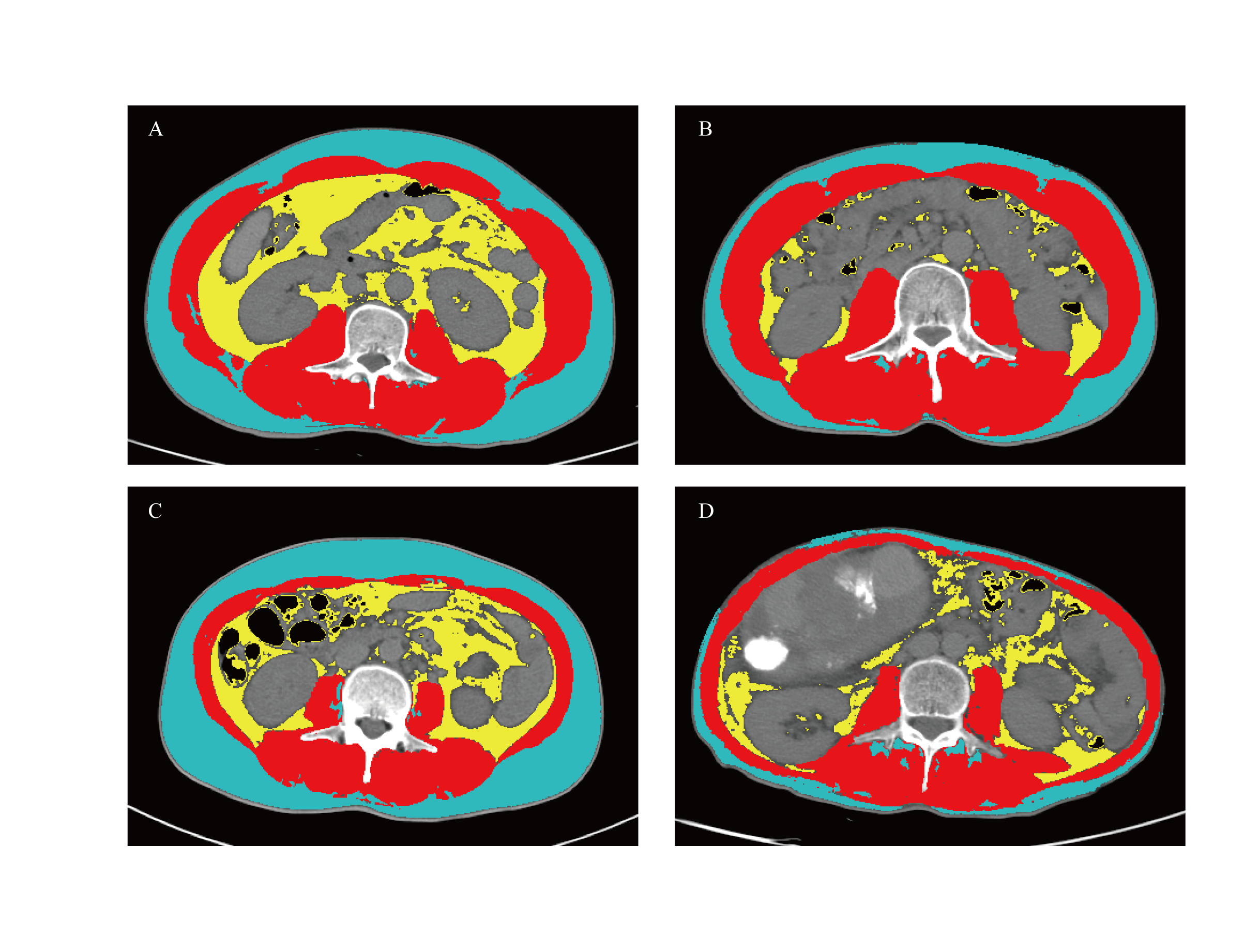

Supplement: Supplementary file 2 — Additional file 2: Fig. S1. Representative segmentation results. We used three colors to label adipose tissue and muscle tissue. Yellow = visceral adipose tissue, Blue = subcutaneous adipose tissue, Red = muscle. [file 12885_2022_9823_MOESM2_ESM.tif]

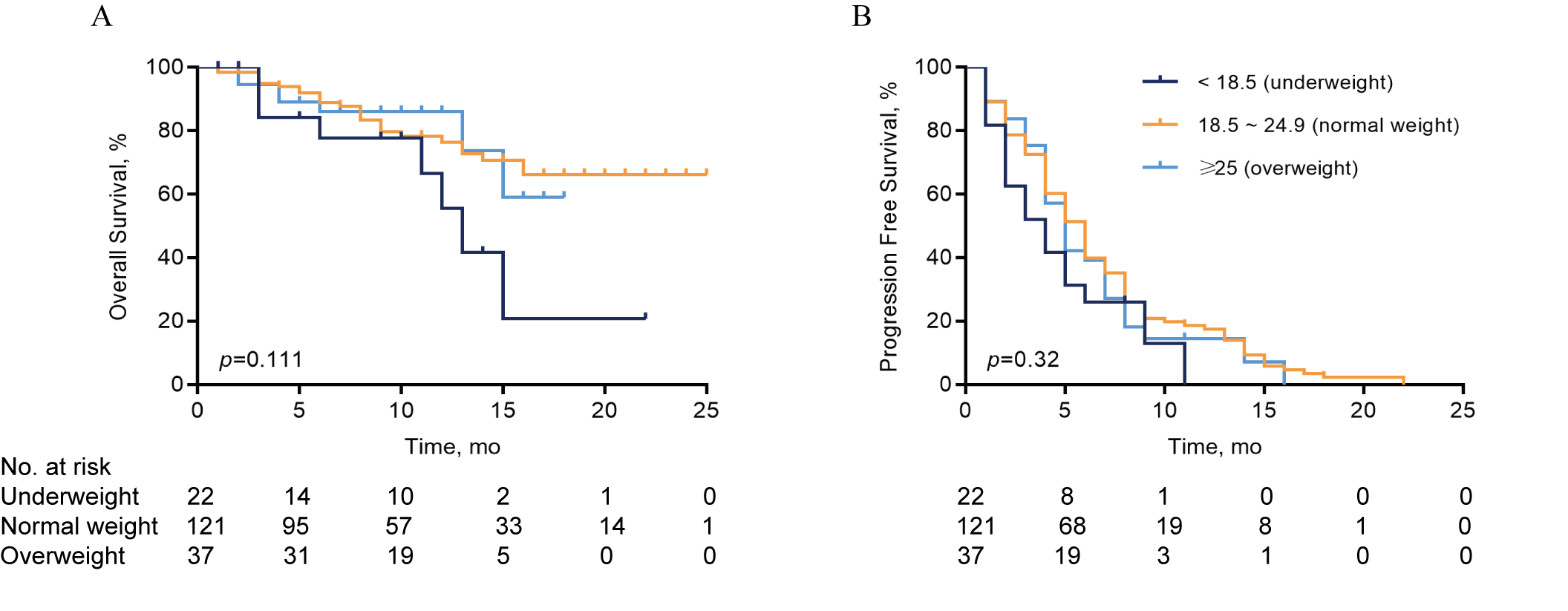

Supplement: Supplementary file 4 — Additional file 4: Fig. S2. Survival outcomes for body mass index. (a) Kaplan- Meier curves for OS. (b) Kaplan- Meier curves for PFS. OS, overall survival; PFS, progression- free survival. The figure showed that the prognosis for underweight patients was worse than that for normal weight and overweight patients; however, the difference was not statistically significant. [file 12885_2022_9823_MOESM4_ESM.tif]

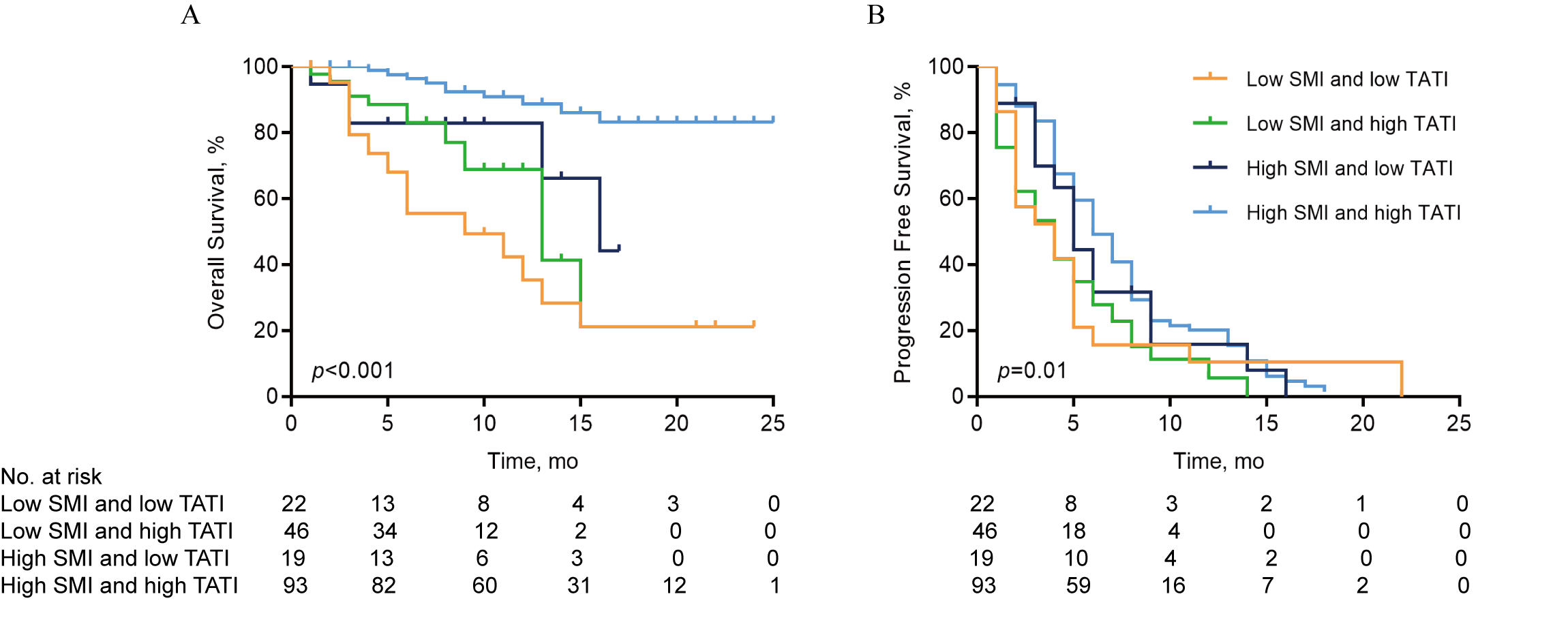

Supplement: Supplementary file 5 — Additional file 5: Fig. S3. Kaplan- Meier curves for PFS and OS for various combinations of SMI:TATI. (a) Kaplan- Meier curves for OS. (b) Kaplan- Meier curves for PFS. OS, overall survival; PFS, progression- free survival SMI, skeletal muscle index; TATI, total adipose tissue index. We compared cohorts with the poorest outcomes (low SMI:low TATI) to those with the best outcomes (high SMI:high TATI) and found that there was a significant difference in both PFS and OS with patients in the low SMI:low TATI group having significantly worse outcomes (p < 0.001 and p = 0.020 respectively). [file 12885_2022_9823_MOESM5_ESM.tif]
