# Supplementary material for: Use of computed tomography-derived body composition to determine the prognosis of patients with primary liver cancer treated with immune checkpoint inhibitors: a retrospective cohort study
Source: BMC Cancer. 2022 Jul 6;22:737. doi: 10.1186/s12885-022-09823-7 (PMC9258103; doi:10.1186/s12885-022-09823-7)
Supplement: Supplementary file 3 — Additional file 3: Table S2. Univariable and multivariable analysis examining OS, and PFS in association with BMI. The univariate and multivariate analyses did not indicate any significant differences between BMI and OS, or PFS. [file 12885_2022_9823_MOESM3_ESM.docx]

**Additional file 3.**

**Table S2. Univariable and multivariable analysis examining OS, and PFS in association with BMI (n=172)**

| **Univariable analysis** | | | |
| --- | --- | --- | --- |
| **OS** | **HR** | **95% CI** | **P value** |
| Underweight | 2.13 | (0.96-4.75) | 0.063 |
| Normal weight | 1.00 | (referent) |  |
| Obesity | 0.92 | (0.40-2.12) | 0.839 |
| **PFS** | **HR** | **95% CI** | **P value** |
| Underweight | 1.37 | (0.83-2.28) | 0.220 |
| Normal weight | 1.00 | (referent) |  |
| Obesity | 1.06 | (0.71-1.59) | 0.766 |
| **Multivariable analysis*** | | | |
| **OS** | **HR** | **95% CI** | **P value** |
| Underweight | 1.95 | (0.81-4.69) | 0.134 |
| Normal weight | 1.00 | (referent) |  |
| Obesity | 1.06 | (0.45-2.52) | 0.888 |
| **PFS** | **HR** | **95% CI** | **P value** |
| Underweight | 1.39 | (0.81-2.38) | 0.231 |
| Normal weight | 1.00 | (referent) |  |
| Obesity | 1.11 | (0.73-1.69) | 0.623 |

OS, overall survival; PFS, progression- free survival; BMI, body mass index; HR, hazard ratio; CI, confidence interval.

*Adjusted for age, sex, BCLC stage, ECOG-PS, Child-Pugh Class, baseline metastasis and previous treatment.
