# Supplementary material for: Use of computed tomography-derived body composition to determine the prognosis of patients with primary liver cancer treated with immune checkpoint inhibitors: a retrospective cohort study
Source: BMC Cancer. 2022 Jul 6;22:737. doi: 10.1186/s12885-022-09823-7 (PMC9258103; doi:10.1186/s12885-022-09823-7)
Supplement: Supplementary file 6 — Additional file 6. STROBE Statement—checklist of items that should be included in reports of observational studies. [file 12885_2022_9823_MOESM6_ESM.docx]

STROBE Statement—checklist of items that should be included in reports of observational studies

|  | Item No. | Recommendation | Page  No. | | | | Relevant text from manuscript |  |
| --- | --- | --- | --- | --- | --- | --- | --- | --- |
| **Title and abstract** | 1 | (*a*) Indicate the study’s design with a commonly used term in the title or the abstract | Page 3/line 30-34 | | | | Abstract/Para 2 |  |
|  |  | (*b*) Provide in the abstract an informative and balanced summary of what was done and what was found | Page 3/line 30-39 | | | | Abstract/Para 2-3 |  |
| Introduction | | | | | | |  |  |
| Background/rationale | 2 | Explain the scientific background and rationale for the investigation being reported | Page 4/line 45-65 | | | | Background/Para 1-2 |  |
| Objectives | 3 | State specific objectives, including any prespecified hypotheses | Page 4/line 63-65 | | | | Background/Para 2 |  |
| Methods | | | | | | |  |  |
| Study design | 4 | Present key elements of study design early in the paper | Page 5/line 69-81 | | | | Patient population/Para 1 |  |
| Setting | 5 | Describe the setting, locations, and relevant dates, including periods of recruitment, exposure, follow-up, and data collection | Page 5/line 69-81 | | | | Patient population/Para 1 |  |
| Participants | 6 | (*a*) *Cohort study*—Give the eligibility criteria, and the sources and methods of selection of participants. Describe methods of follow-up  *Case-control study*—Give the eligibility criteria, and the sources and methods of case ascertainment and control selection. Give the rationale for the choice of cases and controls  *Cross-sectional study*—Give the eligibility criteria, and the sources and methods of selection of participants | Page 5/line 69-75 | | | | Patient population/Para 1 |  |
|  |  | (*b*) *Cohort study*—For matched studies, give matching criteria and number of exposed and unexposed  *Case-control study*—For matched studies, give matching criteria and the number of controls per case | N/A (We have not used a matched analysis in this study.) | | | | N/A (We have not used a matched analysis in this study.) |  |
| Variables | 7 | Clearly define all outcomes, exposures, predictors, potential confounders, and effect modifiers. Give diagnostic criteria, if applicable | Page 5-7/line 76-123 | | | | Patient population/Para 1  Automatic segmentation/Para 1  Body composition measurements/Para 1  Body mass index/Para 1 |  |
| Data sources/ measurement | 8* | For each variable of interest, give sources of data and details of methods of assessment (measurement). Describe comparability of assessment methods if there is more than one group | Page 5-7/line 76-123 | | | | Patient population/Para 1  Automatic segmentation/Para 1  Body composition measurements/Para 1  Body mass index/Para 1 |  |
| Bias | 9 | Describe any efforts to address potential sources of bias | Page 5-6/line 83-98 | | | | Automatic segmentation/Para 1 |  |
| Study size | 10 | Explain how the study size was arrived at | Page 5/line 69-75 | | | | Patient population/Para 1 |  |
| Quantitative variables | 11 | Explain how quantitative variables were handled in the analyses. If applicable, describe which groupings were chosen and why | Page 6-7/line 99-123 | | | Body composition measurements/Para 1  Body mass index/Para 1 | |  |
| Statistical methods | 12 | (*a*) Describe all statistical methods, including those used to control for confounding | Page 7-8/line 124-136 | | | Statistical analysis/Para 1 | |  |
|  |  | (*b*) Describe any methods used to examine subgroups and interactions | N/A(no subgroup analysis was performed) | | | N/A(no subgroup analysis was performed) | |  |
|  |  | (*c*) Explain how missing data were addressed | N/A(no missing data) | | | N/A(no missing data) | |  |
|  |  | (*d*) *Cohort study*—If applicable, explain how loss to follow-up was addressed  *Case-control study*—If applicable, explain how matching of cases and controls was addressed  *Cross-sectional study*—If applicable, describe analytical methods taking account of sampling strategy | Page 5/line 77-79 | | | Patient population/Para 1 | |  |
|  |  | (*e*) Describe any sensitivity analyses | N/A(no sensitivity analyses) | | | N/A(no sensitivity analyses) | |  |
| Results | | | | | | | |  |
| Participants | 13* | (a) Report numbers of individuals at each stage of study—eg numbers potentially eligible, examined for eligibility, confirmed eligible, included in the study, completing follow-up, and analysed | Page 8/line 140-144 | | | Patient characteristics/Para 1 | |  |
|  |  | (b) Give reasons for non-participation at each stage | Page 8/line 140-144 | | | Patient characteristics/Para 1 | |  |
|  |  | (c) Consider use of a flow diagram | Page 5/line 75 | | | Patient population/Para 1 | |  |
| Descriptive data | 14* | (a) Give characteristics of study participants (eg demographic, clinical, social) and information on exposures and potential confounders | Page 8/line 145-149 | | | Patient characteristics/Para 2 | |  |
|  |  | (b) Indicate number of participants with missing data for each variable of interest | N/A(no missing data) | | | N/A(no missing data) | |  |
|  |  | (c) *Cohort study*—Summarise follow-up time (eg, average and total amount) | Page 8/line 148-149 | | | Patient characteristics/Para 2 | |  |
| Outcome data | 15* | *Cohort study*—Report numbers of outcome events or summary measures over time | N/A | | | N/A | |  |
|  |  | *Case-control study—*Report numbers in each exposure category, or summary measures of exposure | N/A(not case-control study) | | | N/A(not case-control study) | |  |
|  |  | *Cross-sectional study—*Report numbers of outcome events or summary measures | N/A(not cross-sectional study) | | | N/A(not cross-sectional study) | |  |
| Main results | 16 | (*a*) Give unadjusted estimates and, if applicable, confounder-adjusted estimates and their precision (eg, 95% confidence interval). Make clear which confounders were adjusted for and why they were included | Page 9-10/line 157-182 | | | Associations with body mass index/Para 1  Association with body composition measures/Para 1-2 | |  |
|  |  | (*b*) Report category boundaries when continuous variables were categorized | Page 9-10/line 157-182 | | | Associations with body mass index/Para 1  Association with body composition measures/Para 1-2 | |  |
|  |  | (*c*) If relevant, consider translating estimates of relative risk into absolute risk for a meaningful time period | N/A(we have used HR) | | | N/A(we have used HR) | |  |
| Other analyses | 17 | Report other analyses done—eg analyses of subgroups and interactions, and sensitivity analyses | N/A(no other analyses) | | | N/A(no other analyses) | |  |
| Discussion | | | | | | | |  |
| Key results | 18 | Summarise key results with reference to study objectives | Page 13/line 232-237 | | | Conclusions/Para 1 | |  |
| Limitations | 19 | Discuss limitations of the study, taking into account sources of potential bias or imprecision. Discuss both direction and magnitude of any potential bias | Page 12/line 224-229 | | | Discussion/Para 4 | |  |
| Interpretation | 20 | Give a cautious overall interpretation of results considering objectives, limitations, multiplicity of analyses, results from similar studies, and other relevant evidence | | Page 11-12/line 192-223 | | Discussion/Para 1-3 | | |
| Generalisability | 21 | Discuss the generalisability (external validity) of the study results | N/A | | N/A | | |  |
| Other information | |  | | | | | |  |
| Funding | 22 | Give the source of funding and the role of the funders for the present study and, if applicable, for the original study on which the present article is based | Page 14/line 266-270 | | Funding/Para 1 | | |  |

*Give information separately for cases and controls in case-control studies and, if applicable, for exposed and unexposed groups in cohort and cross-sectional studies.

**Note:** An Explanation and Elaboration article discusses each checklist item and gives methodological background and published examples of transparent reporting. The STROBE checklist is best used in conjunction with this article (freely available on the Web sites of PLoS Medicine at http://www.plosmedicine.org/, Annals of Internal Medicine at http://www.annals.org/, and Epidemiology at http://www.epidem.com/). Information on the STROBE Initiative is available at www.strobe-statement.org.
